# Mental health, physical health, and health-related behaviors of U.S. Army Special Forces

**DOI:** 10.1371/journal.pone.0233560

**Published:** 2020-06-03

**Authors:** Adam D. Cooper, Steven G. Warner, Anna C. Rivera, Rudolph P. Rull, Amy B. Adler, Dennis J. Faix, Rob Neff, Edwin A. Deagle, Ryan J. Caserta, Cynthia A. LeardMann, for the Millennium Cohort Study Team

**Affiliations:** 1 Deployment Health Research Department, Naval Health Research Center, San Diego, CA, United States of America; 2 Warfighter Performance Department, Naval Health Research Center, San Diego, CA, United States of America; 3 Innovative Employee Solutions, San Diego, CA, United States of America; 4 Leidos, Reston, VA, United States of America; 5 Walter Reed Army Institute of Research, Silver Spring, MD, United States of America; 6 Naval Medical Research Unit Two, Phnom Penh, Cambodia; 7 Preservation of the Force and Family, Headquarters United States Special Operation Command, MacDill Air Force Base, Tampa, FL, United States of America; University of Pittsburgh, UNITED STATES

## Abstract

**Objectives:**

To prospectively examine the health and health-related behaviors of Army Special Forces personnel in comparison with two distinct, but functionally similar Army groups.

**Methods:**

Special Forces, Ranger Qualified, and General Purposes Forces enrolled in the Millennium Cohort Study were identified using data from the Defense Manpower Data Center. Using prospective survey data (2001–2014), we estimated the association of Army specialization with mental health, social support, physical health, and health-related behaviors with multivariable regression models.

**Results:**

Among the 5,392 eligible participants (84.4% General Purposes Forces, 10.0% Special Forces, 5.6% Ranger Qualified), Special Forces personnel reported the lowest prevalence of mental disorders, physical health problems, and unhealthy behaviors. In the multivariable models, Special Forces personnel were less likely to report mental health problems, multiple somatic symptoms, and unhealthy behaviors compared with General Purpose Forces infantrymen (odds ratios [OR]: 0.20–0.54, p-values < .01). Overall, Special Forces personnel were similar in terms of mental and physical health compared with Ranger Qualified infantrymen, but were less likely to sleep < 5 hours/night (OR: 0.60, 95% confidence intervals: 0.40, 0.92) and have 5 or more multiple somatic symptoms (OR: 0.69, 95% CI: 0.49, 0.98). Both Special Forces personnel and Ranger Qualified infantrymen engaged in more healthy behaviors compared with General Purpose Forces infantrymen (OR: 2.57–6.22, p-values<0.05). Engagement in more healthy behaviors reduced the odds of subsequent adverse health outcomes, regardless of specialization.

**Conclusions:**

Army Special Forces personnel were found to be mentally and physically healthier than General Purpose Forces infantrymen, which may in part be due to their tendency to engage in healthy behaviors. Findings indicate that engagement in a greater number of healthy behaviors may reduce odds for subsequent adverse outcomes.

## Introduction

The U.S. Army Special Forces (SF) are elite cohesive units expected to perform in high-risk operational contexts. Because missions carried out by these individuals often involve extreme levels of stress and fierce combat spanning months at a time, SF personnel must pass multi-faceted screenings and undergo rigorous training to ensure they are qualified and adequately prepared. SF personnel are often the first service members deployed to remote and dangerous locations and thus experience more frequent and intense combat deployments than non-SF personnel [1–4]. They may thus be predisposed to a higher likelihood of mental and physical health problems [5]. The majority of previous studies, however, suggested that SF personnel were less likely to exhibit symptoms or seek medical care for mental and physical health problems compared with those from other military occupational populations, including general infantry, combat artillery, and mechanized infantry soldiers [6–10].

Numerous factors likely contribute to the individuals, who are selected and serve as SF personnel, being healthier and more resilient. They complete intense screening and training prior to and between deployments and serve in tight-knit cohesive groups that can take pride in their expertise and elite status [11]. While each of these factors may play a role in the resilience and hardiness of SF individuals, SF personnel may also engage in more healthy behaviors such as sleeping 7 to 9 hours most nights and higher levels of physical activity [9, 12]. In civilian populations, engagement in healthy behaviors (e.g., adequate sleep, regular exercise) have been associated with better mental health [13–19], a reduction of chronic pain [20–22], and overall well-being [23–25].

Despite previous studies reporting that SF personnel were healthier than non-SF personnel, these analyses were limited by cross-sectional designs [6,7], unavailability of data on potential confounding factors such as combat history [7, 8, 9], and/or an inability to differentiate the roles of personnel serving in SF units (e.g., SF, support, medical) [6, 8, 9]. Furthermore, previous investigations have not identified the specific factors that explain why SF personnel may exhibit more positive mental and physical health outcomes than non-SF personnel.

To our knowledge, the current study is the first to evaluate and compare mental health, social support, physical health, and health-related behaviors among SF personnel and other similar occupational specializations using data from a longitudinal study. This approach may provide critical insights toward understanding the strengthening aspects of selection, training, experience, unit culture, and health-related behaviors.

The first aim of this study was to compare the mental health, physical health, social support, and health-related behaviors of SF personnel with two functionally similar Army groups (General Purpose Forces (GPF) and Ranger Qualified infantrymen (RQ)), while adjusting for baseline health, demographics, and military factors. The second aim compared the number of healthy behaviors between specializations. Finally, we examined the association of number of healthy behaviors with subsequent health outcomes, and whether this association varied by military specialization.

## Methods

### Study population

The Millennium Cohort Study was established in 2001 to prospectively investigate and examine the long-term impact of military service. Invited personnel were randomly sampled from U.S. military rosters, representing all service branches and components, with oversampling of selected subgroups, as previously described [26]. Participants were enrolled and completed their first survey in four separate phases between 2001 and 2014. Participants were then requested to complete additional surveys approximately every 3–4 years, including after their separation from military service. The surveys assessed participants’ mental health, physical health, and health-related behaviors, as well as military and non-military experiences [27–28]. The Millennium Cohort procedures were approved by the Institutional Review Board at the Naval Health Research Center (NHRC 2000.0007). All participants provided written voluntary informed consent.

For this study, SF personnel were compared with two other types of Army soldiers who also serve in combat roles: RQ and GPF infantrymen. SF personnel consisted of those who had passed the 24-day Special Forces Assessment and Selection Course followed by the 12–18 months Special Forces Qualification Course. Many of these individuals have also completed Ranger school and usually serve in small units with other SF personnel. RQ infantrymen consisted of infantry soldiers who volunteered for and completed Ranger school, the U.S. military’s 9-week premier leadership course. After completing this leadership course, most serve in conventional Army units and are not considered to be members of the Special Operations Forces. GPF infantrymen included Soldiers who met Army requirements for this military occupational specialty in terms of mandatory training, cognitive scores, and physical fitness standards.

Type of Army specialization was assessed using Military Occupational Specialty (MOS) and identifiers obtained from the Defense Manpower Data Center (DMDC) between 2001 and 2014. SF personnel were defined as those who had an MOS code beginning with 18B, 18C, 18D, 18E, 18F, or 18Z (Weapons Sergeant, Engineer Sergeant, Medical Sergeant, etc.) for enlisted personnel and 180A or 18A for commissioned and warrant officers, respectively. RQ infantrymen were defined as individuals who had completed Ranger School and were awarded the ‘Ranger Tab.’ Enlisted RQ infantrymen had an MOS beginning with 11B, 11C, 11H, 11M, or 11Z (Infantryman–Ranger Parachutist, Indirect Fire Infantryman–Ranger, Infantry Senior Sergeant–Ranger, etc.) and a Special Qualification Identifier of G or V. RQ officers had an MOS beginning with 11A and an additional skills identifier of 5R or 5S. GPF infantrymen had an MOS beginning with 11A, 11B, 11C, 11H, 11M, or 11Z (Infantry Officer (Commissioned), Infantryman, Indirect Fire Infantryman, Heavy Antiarmor Weapons Infantryman, etc.), but did not have the additional required identifiers to be categorized as RQ. To ensure homogenous groups, Soldiers identified as ever serving in the 75^th^ Ranger Regiment unit, a Special Operations Force, were excluded from all analyses.

The sample for this study was restricted to Millennium Cohort male participants from any of the four enrollment cycles who were SF personnel, RQ infantrymen, or GPF infantrymen (*n* = 10,116). In order to conduct a prospective analysis, participants must have completed two consecutive surveys, with at least one completed while serving in the military in their specialization (i.e., SF personnel, RQ infantrymen, or GPF infantrymen) (excluded *n* = 4,586). The first of these two consecutive surveys was referred to as “baseline” and the second as “follow-up.” Of the 5,530 eligible participants, those who did not complete demographic, military, and baseline health data were excluded (*n* = 138), thus yielding a final study population of 5,392 soldiers.

### Measures

Mental health, physical health, social support, and health-related behaviors were assessed using data from the follow-up survey. The four mental health outcomes included posttraumatic stress disorder (PTSD), depression, panic/anxiety, and an aggregated outcome of the four disorders referred to as “any mental disorder.” PTSD was assessed using the standardized PTSD Checklist–Civilian Version (PCL-C) (Cronbach’s α = 0.97). Based on the Diagnostic and Statistical manual of Mental Disorders, 4th edition (DSM-IV) diagnostic criteria (i.e.,endorsing “moderately” or higher on one or more intrusion items, two or more hyper-arousal items, and three or more avoidance items in the past month), which has demonstrated strong psychometric properties, participants were classified as screening positive for PTSD [29]. Standardized items and scoring of the Primary Care Evaluation of Mental Disorders Patient Health Questionnaire (PHQ) were used to assess depression, panic syndrome, and other anxiety syndromes. Previous studies observed good agreement between PHQ diagnoses and those of mental health professionals [30]. Using the DSM-IV major depressive disorder criteria [31], individuals who endorsed five of the eight PHQ items (i.e., “more than half the days” or higher), including anhedonia or depressed mood were identified as screening positive for depression (Cronbach’s α = 0.92). Participants were identified as having panic syndrome if they positively responded to specific psychosocial questions and had four or more symptoms of an anxiety attack. Other anxiety syndrome was measured using six items from the PHQ. Participants were classified as screening positive for panic/anxiety if they were positive for panic syndrome and/or other anxiety syndrome [32].

Social support was assessed using one PHQ item. Individuals reporting being “bothered a lot” or “bothered a little” by “having no one to turn to when you have a problem” in the last 4 weeks were defined as having a lack of social support.

The two physical health outcomes were bodily pain and somatic symptoms. Bodily pain was based on the two pain items from the Medical Outcomes Short Form 36-Item Health Survey for Veterans (SF-36V). Using the standardized scoring algorithm of the SF-36V scales, the responses were summed and scored with a range from 0 to 100. Bodily pain was further categorized into tertiles as low, medium, and high bodily pain [33–35]. Somatic symptoms were based on 14 PHQ items that assess physical health symptoms (e.g., headaches, dizziness, nausea) in the last 4 weeks (Cronbach’s α = 0.85). The total number of symptoms were summed based on standardized scoring and then categorized as 0–4 or 5+ symptoms [32].

Health-related behaviors consisted of alcohol-related problems, smoking, sleep hours, trouble sleeping, physical activity, and fast food consumption. Alcohol-related problems was defined as reporting at least one of the five alcohol-related PHQ items in the last 12 months (Cronbach’s α = 0.68) [30]. Smoking behavior was categorized as non-smokers (<100 cigarettes/lifetime), former smokers (≥100 cigarettes/lifetime, successfully quit), and current smokers (≥100 cigarettes/lifetime, had not successfully quit). Sleep hours was assessed using the number of hours of sleep in an average 24-hour period in the last month. Trouble sleeping was defined as reporting “trouble falling asleep or staying asleep” in the last month on either the PCL-C (“moderately” or more) or the PHQ (“several days” or more) item [36]. Physical activity level was ascertained from the self-reported number of minutes engaged in activity per week and categorized as low (cannot physically do, or <150 minutes), medium (150–299 minutes), medium-high (300–449 minutes per week), and high (≥450 minutes) [37]. Fast food consumption was categorized as “None,” “Once per week,” “2–3 times per week,” and “4+ times per week.”

For the second aim, the number of healthy behaviors was based on the total number of healthy behaviors endorsed at baseline. Using the same algorithm as previous studies, each of the six behaviors was categorized as “healthy,” and then summed (i.e., score 0–6) [38]. The healthy behaviors included lower alcohol consumption (1–14 drinks/week), not smoking (i.e., non-smoker), adequate sleep (7–9 hrs/day), physical activity (≥150 minutes of weekly physical activity), low sedentary lifestyle factors (less than 8 hours/day), and low fast food consumption (<1 time/week) [38]. Based on low frequencies for the lowest and highest number of healthy behaviors, this variable was categorized as 0–2, 3, 4, and 5–6 healthy behaviors.

### Covariates

Covariates were assessed at baseline using data obtained from the survey and the DMDC. Educational level and marital status were assessed using self-reported survey data. Deployment experience was based on deploying prior to baseline, either self-reported or based on an electronic record in the Contingency Tracking System, in conjunction with baseline items that assessed combat experiences. Participants who had at least one deployment prior to baseline were classified as “deployed without combat” or “deployed with combat,” based on endorsement of one of more combat items. Participants who did not deploy prior to baseline were categorized as “not deployed.” Baseline mental component scores and physical component scores were assessed and calculated using the standard items and scoring from the SF-36V [33–35], where higher scores indicate better health. All other demographic (e.g., race/ethnicity, age) and military service characteristics (e.g., service component, pay grades) were determined using DMDC records.

### Statistical analysis

Descriptive statistics assessed the distributions of demographic characteristics, military factors, baseline health, and outcomes by type of specialization. For the primary aim, multivariable logistic regression was used to estimate the association between specialization and each mental health, social support, physical health, and health-related behavior outcome, with separate models run for each outcome. Models were adjusted for demographics, military factors, and baseline health. Analyses where SF personnel were the occupational category of interest utilized distinct reference groups, GPF infantrymen and RQ infantrymen, in separate models.

The second aim examined the number of healthy behaviors. Because three of the behaviors (fast food, sedentary lifestyle, and physical activity) were not assessed at baseline for the first two enrollment panels, those participants who completed baseline surveys without these items (*n* = 3,000), along with those missing any of the six assessed behaviors (*n* = 120), were excluded, thus reducing the study population for these analyses to 2,272 participants. The relationship between specialization and baseline number of healthy behaviors was assessed using multivariable logistic regression.

For the third aim, we estimated the association between the baseline number of healthy behaviors and subsequent health outcomes (e.g., any mental disorder, bodily pain). Limiting the health outcomes of interest was necessary due to the inclusion of some of the behaviors in the healthy behaviors predictor variable. Logistic regression models were adjusted for demographics, military factors, and baseline health. To assess whether the association between the number of healthy behaviors and health outcomes was modified by type of specialization, we included an interaction term (specialization * healthy behaviors).

Since behaviors and health status may change upon leaving military service, we conducted sub-analyses in which we excluded those who were no longer serving in the military at the time of their follow-up survey, resulting in the removal of 23% of the study population. All models in Tables 3, 4 and 5 were re-analyzed with this restricted population, with the exception of the specific mental health disorders, PTSD, depression, panic/anxiety, due to low numbers. In addition, we conducted analyses where we adjusted for time between surveys.

For all models, collinearity was assessed and defined as a value >4 using the variance inflation factor. Data management and all statistical analyses were conducted using SAS (version 9.4, SAS Institute, Inc., Cary, NC, USA).

## Results

Most of the 5,392 study participants were GPF infantrymen (84.4%), while 10.0% were SF personnel, and 5.6% were RQ infantrymen. SF personnel were proportionally more likely to be non-Hispanic white, higher educated, and older compared with GPF and RQ infantrymen (Table 1). In addition, SF personnel were proportionally more likely to be married, serving on active duty, and an officer than GPF personnel. At baseline, 70.8% of SF personnel had been previously deployed and experienced combat compared with 66.0% for RQ infantrymen and 58.4% for GPF infantrymen. SF personnel were proportionally more likely to report higher levels of physical and mental functioning at baseline than GPF and RQ infantrymen (Table 1). On average, GPF infantrymen had served the longest in their specialization at baseline (71.1 months, SD: 30.0), followed by RQ (52.2 months, SD: 25.5) and SF personnel (47.7 months, SD: 28.4). The mean number of months between baseline and follow-up surveys for all groups was similar (35.4–37.5 months).

**Table 1:** Baseline characteristics of soldiers by Army specialization, the Millennium Cohort Study*.

	General Purpose Forces (*n* = 4,552) *n* (%)	Ranger Qualified (*n* = 303) *n* (%)	Special Forces (*n* = 537) *n* (%)
Demographics			
**Race/ethnicity**			
Asian/Pacific Islander	176 (3.9)	10 (3.3)	19 (3.5)
Black, non-Hispanic	255 (5.6)	21 (6.9)	13 (2.4)
White, non-Hispanic	3,703 (81.4)	247 (81.5)	479 (89.2)
Hispanic	326 (7.2)	18 (5.9)	18 (3.4)
Other	92 (2.0)	7 (2.3)	8 (1.5)
**Marital status**			
Married	2,768 (60.8)	208 (68.7)	355 (66.1)
Not married	1,784 (39.2)	95 (31.4)	182 (33.9)
**Education**			
High school degree or less	1,107 (24.3)	30 (9.9)	32 (6.0)
Some college, no degree	1,759 (38.6)	101 (33.3)	169 (31.5)
Associate’s degree	321 (7.1)	20 (6.6)	52 (9.7)
Bachelor’s degree	1,050 (23.1)	116 (38.3)	215 (40.0)
Master’s degree or higher	315 (6.9)	36 (11.9)	69 (12.9)
**Age (years)**			
17–24	1,322 (29.0)	48 (15.8)	70 (13.0)
25–34	2,022 (44.4)	161 (53.1)	281 (52.3)
35–44	911 (20.0)	81 (26.7)	149 (27.8)
44+	297 (6.5)	13 (4.3)	37 (6.9)
Military Service			
**Service component**			
Active duty	2,374 (52.2)	246 (81.2)	427 (79.5)
Reserves	2,178 (47.9)	57 (18.8)	110 (20.5)
**Pay grade**			
Enlisted	3,674 (80.7)	172 (56.8)	340 (63.3)
Officer	878 (19.3)	131 (43.2)	197 (36.7)
**Recent deployment experience**^a^			
Not deployed	1,328 (29.2)	71 (23.4)	94 (17.5)
Deployed, no combat	566 (12.4)	32 (10.6)	63 (11.7)
Deployed with combat	2,658 (58.4)	200 (66.0)	380 (70.8)
Baseline Health^b^			
**Mental functioning**			
High	624 (13.7)	59 (19.5)	126 (23.5)
Medium	3,182 (69.9)	215 (71.0)	378 (70.4)
Low	746 (16.4)	29 (9.6)	33 (6.2)
**Physical functioning**			
High	660 (14.5)	56 (18.5)	93 (17.3)
Medium	3,165 (69.5)	211 (69.6)	399 (74.3)
Low	727 (16.0)	36 (11.9)	45 (8.4)

*All characteristics differed significantly (p<0.05) by specialization.

^a^Based on deployment in support of Operation Iraqi Freedom, Operation Enduring Freedom, or Operation New Dawn and self-report of combat experiences prior to baseline.

^b^Based on mental component score and physical component score, which have been categorized as low (<15^th^), medium (15^th^ to 85^th^), and high (>85^th^).

With the exception of depression, SF personnel reported the lowest frequencies of mental and physical health problems at follow-up, followed by RQ infantrymen, and then GPF infantrymen (Table 2). Depression frequencies were similar for SF personnel and RQ infantrymen, but higher for GPF infantrymen. SF personnel (40.3%) were proportionally more likely to report 5–6 healthy behaviors compared with RQ infantrymen (26.9%) and GPF infantrymen (19.1%).

**Table 2:** Frequencies of mental health, social support, physical health, health-related behaviors, and number of healthy behaviors by Army specialization among soldiers, the Millennium Cohort Study*.

	General Purpose Forces *n* = 4,552 *n* (%)^a^	Ranger Qualified *n* = 303 *n* (%)^a^	Special Forces *n* = 537 *n* (%)^a^
Mental Health			
**PTSD**^b^	625 (13.7)	11 (3.6)	12 (2.2)
**Depression**^c^	354 (7.8)	4 (1.3)	8 (1.5)
**Panic/anxiety**^d^	403 (8.9)	8 (2.6)	6 (1.1)
**Any mental disorder**^e^	763 (16.8)	16 (5.3)	17 (3.2)
Social Support			
**Lack of social support**^f^	1,086 (23.9)	46 (15.2)	53 (9.9)
Physical Health			
**Bodily Pain**^g^			
Low	1,850 (40.6)	147 (48.5)	243 (45.3)
Medium	1,325 (29.1)	79 (26.1)	159 (29.6)
High	1,315 (28.9)	72 (23.8)	126 (23.5)
**Somatic symptoms**^h^			
0–4 symptoms	2,482 (54.5)	205 (67.7)	398 (74.1)
5+ symptoms	1,875 (41.2)	89 (29.4)	119 (22.2)
Health-related Behaviors			
**Alcohol-related problems**^i^	736 (16.2)	28 (9.2)	38 (7.1)
**Smoking**			
Never smoker	2,220 (48.8)	187 (61.7)	328 (61.1)
Former smoker	1,324 (29.1)	87 (28.7)	160 (29.8)
Current smoker	822 (18.1)	20 (6.6)	25 (4.7)
**Sleep**			
≤ 5 hours	1,251 (27.5)	61 (20.1)	68 (12.7)
6 hours	1,476 (32.4)	99 (32.7)	189 (35.2)
7–9 hours	1,615 (35.5)	131 (43.2)	259 (48.2)
10+ hours	86 (1.9)	5 (1.7)	4 (0.7)
**Trouble sleeping** ^j^	1,803 (39.6)	73 (24.1)	102 (19.0)
**Physical activity**^k^			
High	1,611 (35.4)	131 (43.2)	234 (43.6)
Medium-high	655 (14.4)	54 (17.8)	111 (20.7)
Medium	762 (16.7)	55 (18.2)	95 (17.7)
Low	1,167 (25.6)	41 (13.5)	59 (11.0)
**Fast food consumption**			
None	831 (18.3)	64 (21.1)	118 (22.0)
1 time/wk	1,822 (40.0)	139 (45.9)	222 (41.3)
2–3 times/wk	1,287 (28.3)	71 (23.4)	151 (28.1)
4+ times/wk	414 (9.1)	20 (6.6)	26 (4.8)
Number of Healthy Behaviors^l^			
0–2	353 (18.1)	6 (5.8)	7 (3.2)
3	582 (29.9)	33 (31.7)	51 (23.1)
4	640 (32.9)	37 (35.6)	74 (33.5)
5–6	372 (19.1)	28 (26.9)	89 (40.3)

DSM-IV = Diagnostic and Statistical manual of Mental Disorders, 4th edition; PCL-C = PTSD Check List–Civilian Version; PTSD = posttraumatic stress disorder; PHQ = Patient Health Questionnaire, SF36V = Short Form 36-Item Health Survey for Veterans

*All characteristics differed significantly (p<0.05) by specialization.

^a^Each outcome total and percent varies due to missing data.

^b^PTSD using PCL-C as defined using the DSM-IV criteria.

^c^Based on DSM-IV criteria for major depression (PHQ items)

^d^Using criteria defined by PHQ for panic syndrome and other anxiety syndrome.

^e^Endorsement of PTSD, depression, panic, or anxiety.

^f^ Reporting being “Bothered a lot” or “Bothered a little” to the question “Having no one to turn to when you have a problem,” on PHQ questionnaire.

^g^Based on the bodily pain scale from the SF36V. Categories are based on calculated tertiles.

^h^Based on 14 questions that assess somatic symptoms from the PHQ.

^i^Based on endorsement of at least one item from the PHQ alcohol scale.

^j^Endorsing item “Trouble falling asleep or staying asleep” from either PCL-C or PHQ questionnaire.

^k^Categories are defined as: “Low” is cannot physically do, or <150 minutes per week; “Medium” is 150–299 minutes per week; “Medium-High” is 300–449 minutes per week; and “High” is 450+ minutes per week.

^l^Based on six positive healthy behaviors assessed at baseline (N = 2,272) including: alcohol consumption, smoking, sleep, physical activity, sedentary time, fast food.

Effect estimates for mental health, social support, physical health, and health-related behaviors are listed in Table 3. In models adjusting for demographics, military factors, and baseline health, SF personnel were approximately four times less likely to screen positive for any mental disorder than GPF infantrymen (AOR = 0.25, 95% CI: 0.15, 0.42), while no significant difference was observed when compared with RQ infantrymen. RQ infantrymen were approximately two to three times less likely to screen positive for any mental disorder compared with GPF infantrymen (AOR = 0.38, 95% CI: 0.22, 0.65). Similar associations were observed for the three individual mental health outcomes.

**Table 3:** Adjusted odds ratios of mental health, social support, physical health, and health-related behaviors outcomes by Army specialization among soldiers, the Millennium Cohort Study.

Ou	Special Forces Compared with				Ranger Qualified Compared with General Purpose Forces	
	General Purpose Forces		Ranger Qualified			
	AOR^a^	95% CI	AOR^a^	95% CI	AOR^a^	95% CI
Mental Health						
**PTSD**^**b**^	0.24*	0.13, 0.43	0.66	0.28, 1.54	0.36*	0.19, 0.67
**Depression**^**c**^	0.36*	0.17, 0.74	1.45	0.42, 4.95	0.25*	0.09, 0.68
**Panic/anxiety**^d^	0.20*	0.09, 0.47	0.47	0.16, 1.39	0.43*	0.21, 0.91
**Any mental disorder**^**e**^	0.25*	0.15, 0.42	0.66	0.32, 1.37	0.38*	0.22, 0.65
Social Support						
**Lack of social support**^**f**^	0.50*	0.37, 0.69	0.67	0.43, 1.03	0.75	0.54, 1.06
Physical Health						
**Bodily Pain**^**g**^						
Low	1.00		1.00		1.00	
Medium	0.95	0.76, 1.20	1.25	0.88, 1.76	0.77	0.57, 1.03
High	0.91	0.70, 1.17	1.14	0.78, 1.68	0.79	0.57, 1.10
**Somatic symptoms**^h^						
0–4 symptoms	1.00		1.00		1.00	
5+ symptoms	0.47*	0.37, 0.59	0.69*	0.49, 0.98	0.68*	0.51, 0.90
Health-related Behaviors						
**Alcohol-related problems**^i^	0.54*	0.38, 0.77	0.75	0.44, 1.27	0.72	0.47, 1.08
**Smoking**						
Never smoker	1.00		1.00		1.00	
Former smoker	0.90	0.72, 1.12	0.97	0.70, 1.35	0.92	0.70, 1.22
Current smoker	0.35*	0.23, 0.54	0.75	0.40, 1.41	0.47*	0.29, 0.76
**Sleep**						
≤ 5 hours	0.42*	0.31, 0.56	0.60*	0.40, 0.92	0.69*	0.50, 0.96
6 hours	0.81	0.66, 1.01	1.00	0.72, 1.38	0.81	0.62, 1.08
7–9 hours	1.00		1.00		1.00	
10+ hours	0.57	0.20, 1.64	0.44	0.11, 1.72	1.30	0.50, 3.38
**Trouble sleeping** ^j^	0.39*	0.31, 0.50	0.72	0.51, 1.04	0.54*	0.40, 0.72
**Physical activity**^k^						
High	1.00		1.00		1.00	
Medium-high	1.12	0.86, 1.45	1.20	0.81, 1.77	0.94	0.67, 1.32
Medium	0.87	0.66, 1.14	0.97	0.65, 1.46	0.89	0.64, 1.25
Low	0.47*	0.35, 0.65	0.86	0.54, 1.37	0.55*	0.38, 0.80
**Fast food consumption**						
None	1.00		1.00		1.00	
1 time/wk	0.92	0.72, 1.18	0.88	0.61, 1.28	1.05	0.50, 1.45
2–3 times/wk	1.02	0.78, 1.34	1.21	0.79, 1.83	0.85	0.59, 1.21
4+ times/wk	0.66	0.42, 1.04	0.77	0.40, 1.50	0.85	0.50, 1.45

AOR = Adjusted odds ratio; CI = Confidence Interval; DSM-IV = Diagnostic and Statistical manual of Mental Disorders, 4th edition; SF = Special Forces; PCL-C = PTSD Check List–Civilian Version; PTSD = posttraumatic stress disorder; PHQ = Patient Health Questionnaire, SF36V = Short Form 36-Item Health Survey for Veterans

*p<0.05

^a^Adjusted for race/ethnicity, marital status, education, age, service component, pay grade, recent deployment status, mental health, and physical health.

^b^PTSD using PCL-C as defined using the DSM-IV criteria.

^c^Based on DSM-IV criteria for major depression (PHQ items)

^d^Using criteria defined by PHQ for panic syndrome and other anxiety syndrome.

^e^Endorsement of PTSD, depression, panic, or anxiety.

^f^Reporting being “Bothered a lot” or “Bothered a little” to the question “Having no one to turn to when you have a problem,” on PHQ questionnaire.

^g^Based on the bodily pain scale from the SF36V. Categories are based on calculated tertiles.

^h^Based on the 14 somatic symptoms questions on the PHQ.

^i^Based on endorsement of at least one item from the PHQ alcohol scale.

^j^Endorsing item “Trouble falling asleep or staying asleep” from either PCL-C or PHQ questionnaire.

^k^Categories are defined as: “Low” is cannot physically do, or <150 minutes per week; “Medium” is 150–299 minutes per week; “Medium-High” is 300–449 minutes per week; and “High” is 450+ minutes per week.

With respect to physical health, SF personnel were significantly less likely to report 5+ somatic symptoms compared with GPF infantrymen (AOR = 0.47, 95% CI: 0.37, 0.59) and RQ personnel (AOR = 0.69, 95% CI: 0.49, 0.98). RQ infantrymen were less likely to report 5+ somatic symptoms (AOR = 0.68, 95% CI: 0.51, 0.90) compared with GPF. There were no statistically significant associations for bodily pain between the groups (Table 3).

For social support and health-related behaviors, SF personnel were significantly less likely than GPF infantrymen to report lack of social support (AOR = 0.50, 95% CI: 0.37, 0.69), alcohol-related problems (AOR = 0.54, 95% CI: 0.38, 0.77), current smoking (AOR = 0.35, 95% CI: 0.23, 0.54), sleeping 5 or less hours (AOR = 0.42, 95% CI: 0.31, 0.56), trouble sleeping (AOR = 0.39, 95% CI: 0.31, 0.50), and low levels of physical activity (AOR = 0.47, 95% CI: 0.35, 0.65) (Table 3). There was only one statistically significant difference observed between SF personnel and RQ infantrymen. Specifically, SF personnel were less likely to report sleeping 5 or less hours (AOR = 0.60, 95% CI: 0.40, 0.92) compared with RQ personnel. RQ infantrymen were significantly less likely to report current smoking (AOR = 0.47, 95% CI: 0.29, 0.76), sleeping 5 or less hours (AOR = 0.69, 95% CI: 0.50, 0.96), trouble sleeping (AOR = 0.54, 95% CI: 0.40, 0.72), and low levels of physical activity (AOR = 0.55, 95% CI: 0.38, 0.80) compared with GPF infantrymen. No differences between specializations were observed for the frequency of fast food consumption (Table 3).

The mean number of healthy behaviors was highest for SF personnel (4.2), followed by RQ infantrymen (3.9) and then GPF infantrymen (3.5). Both SF personnel (AOR range: 3.17–6.22) and RQ infantrymen (AOR range = 2.26–2.57) were more likely to report a higher number of healthy behaviors compared with GPF infantrymen, adjusting for baseline health, demographics, and military characteristics (Table 4). However, for SF personnel these associations were statistically significant across all levels, which was not the case for RQ personnel. No significant difference in scores were observed between SF personnel and RQ infantrymen.

**Table 4:** Adjusted odds ratios of composite health score at baseline by Army specialization among soldiers, the Millennium Cohort Study.

Number of healthybehaviors^b^	Special Forces Compared with				Ranger Qualified Compared with	
	General Purpose Forces		Ranger Qualified		General Purpose Forces	
	AOR^a^	95% CI	AOR^a^	95% CI	AOR^a^	95% CI
0–2	1.00		1.00		1.00	
3	3.17*	1.40, 7.20	1.23	0.37, 4.06	2.57*	1.04, 6.38
4	3.45*	1.54, 7.72	1.53	0.47, 4.99	2.26	0.91, 5.60
5–6	6.22*	2.77, 13.97	2.75	0.83, 9.17	2.26	0.88, 5.78

AOR = Adjusted odds ratio

*p<0.05

^a^Adjusted for race/ethnicity, marital status, education, age, service component, pay grade, recent deployment status, mental health, and physical health.

^b^Based on six healthy behaviors including: alcohol consumption, smoking, sleep hours, physical activity, sedentary time, fast food.

Further examination of the relationship between the number of heathly behaviors and the selected outcomes, where all specializations were combined, revealed that those reporting fewer healthy behaviors at baseline had an increased risk for subsequent adverse outcomes (e.g., high bodily pain) (Table 5). Specifically, compared with those reporting 5–6 healthy behaviors at baseline, the risk for a positive screen for any mental disorder increased with each decrement in healthy behaviors (AOR range: 1.73 for 4 behaviors to 2.27 for 0–2 behaviors). A similar pattern was observed for a high level of bodily pain (AOR range: 1.42 for 4 behaviors to 2.26 for 0–2 behaviors) and somatic symptoms (AOR range: 1.31 for 4 behaviors to 1.88 for 0–2 behaviors). Those who reported 0 to 2 baseline healthy behaviors also had an elevated risk for perceived lack of social support (AOR: 2.08, 95% CI: 1.45, 3.00). Tests for heterogeneity did not suggest any interactions between specialization and the composite score for healthy behaviors (p-values: 0.45 to 0.77; results not shown in tables).

**Table 5:** Adjusted odds ratios of outcomes by number of healthy behaviors, all Army specializations combined among soldiers in the Millennium Cohort Study.

Outcome	0–2 health behaviors		3 health behaviors		4 health behaviors	
	compared with 5–6 positive healthy behaviors					
	AOR^a^	95% CI	AOR^a^	95% CI	AOR^a^	95% CI
**Any mental disorder**^b^	2.27*	1.45, 3.55	1.91*	1.27, 2.86	1.73*	1.16, 2.60
**Lack of social support**^c^	2.08*	1.45, 3.00	1.30	0.94, 1.81	1.08	0.78, 1.49
**Bodily Pain**^d^						
Low	1.00		1.00		1.00	
Medium	1.68*	1.15, 2.46	1.26	0.94, 1.70	1.24	0.94, 1.64
High	2.26*	1.50, 3.40	1.55*	1.11, 2.17	1.42*	1.03, 1.96
**Somatic symptoms**^e^						
5+ symptoms reported	1.88*	1.33, 2.64	1.59*	1.20, 2.11	1.31*	1.00, 1.72
0–4 symptoms reported	1.00		1.00		1.00	

AOR = Adjusted odds ratio; PTSD = posttraumatic stress disorder; PHQ = Patient Health Questionnaire, SF36V = Short Form 36-Items Health Survey for Veterans

*p<0.05

^a^Adjusted for race/ethnicity, marital status, education, age, service component, pay grade, mental health, physical health and recent deployment status.

^b^Endorsement of PTSD, depression, panic, or anxiety.

^c^Reporting being “Bothered a lot” or “Bothered a little” to the question “Having no one to turn to when you have a problem,” on PHQ questionnaire.

^d^Based on the bodily pain scale from the SF36V. Categories are based on calculated tertiles.

^e^Based on the 14 somatic symptoms questions on the PHQ.

There were only a few differences in effect estimates when we conducted analyses restricted to those who were still serving in the military at the time of their follow-up survey (n~4,000) (results not shown). Specifically, the magnitude and significance of the association between specialization and all eleven outcomes, including number of healthy behaviors, remained consistent with results from the main analysis (Tables 3 and 4) when comparing SF personnel with GPF infantrymen. Comparing RQ and GPF infantrymen, two associations (somatic symptoms and number of healthy behaviors) were reduced in magnitude and were no longer significant. In comparisons between SF personnel and RQ infantrymen, SF were less likely to report trouble sleeping (OR: 0.66, 95% CI: 0.45–0.97) and lack of social support (OR: 0.59, 95% CI 0.36, 0.95), while the magnitude and significance of the associations for the other outcomes did not appear to change. In the analyses of the association between the number of healthy behaviors and the four outcomes of interest with all specializations combined, the magnitude of association increased for all levels of behavior, except those who reported four healthy behaviors were no longer significantly more likely to report high levels of bodily pain. Adjustment for the time between surveys did not significantly change the effect estimates.

## Discussion

These findings suggest that while SF personnel are known to experience combat deployments of greater intensity, they also appear to be the healthiest of the three groups examined. SF personnel were less likely than GPF infantrymen to report psychological health disorders, somatic symptoms, unhealthy behaviors (e.g., alcohol-related problems, smoking, trouble sleeping), and more likely to report social support and healthy behaviors (e.g., sleeping 7–9 hours, high levels of physical activity). Both SF personnel and RQ infantrymen reported engaging in a higher number of healthy behaviors compared with GPF infantrymen. Engagement in more healthy behaviors was associated with decreased risk for subsequent adverse health outcomes, regardless of Army specialization.

These results corroborate and expand upon previous findings indicating that SF personnel have better mental health and physical health compared with other soldiers [7–9]. One novel finding was of similarities in mental health and physical health between SF personnel and RQ infantrymen. For these select groups, these lower frequencies of adverse mental health outcomes and somatic symptoms in relation to GPF infantrymen may be attributable to innate characteristics of those who strive for and successfully complete these elite assignments and trainings.

Our results suggest that differences observed in mental and physical health outcomes between SF personnel and RQ infantrymen compared with GPF infantrymen may also be partly attributable to engagement in healthy behaviors. We found that SF personnel and RQ infantrymen were more likely to engage in a greater number of healthy behaviors and less likely to engage in unhealthy behaviors compared with GPF infantrymen. Moreover, the number of healthy behaviors at baseline was associated with social support and health outcomes at follow-up. These observed links between healthy behaviors and better mental and physical health are consistent with results from other populations [13–22].

SF personnel reported better sleep than both GPF and RQ infantrymen. Unlike GPF and RQ infantrymen, SF personnel serve together under one command (United States Army Special Operations Command) and therefore may receive more consistent guidance on the importance of healthy sleep patterns [11]. In contrast, GPF and RQ infantrymen are assigned to a wide range of Army commands. These various commands may provide inconsistent guidance and unit culture regarding healthy behaviors, with some placing greater importance on sleep than others, potentially due to differing mission demands, requirements, leadership emphasis, or unit norms.

While the findings from the current study suggest engagement of healthy behaviors and reduction of unhealthy behaviors may benefit all soldiers regardless of specialization, a pertinent question for future research remains how to facilitate the widespread implementation and adoption of these behaviors. The two general approaches are either to treat the already established unhealthy behaviors with therapy, education, and/or pharmacological programs, or to develop a proactive approach to prevent them from occurring in the first place. Treatments to address tobacco use and insomnia, for example, have had measureable success in modifying behaviors [39–43]. However, a proactive approach would be more efficient and potentially impactful [44–46]. The Army’s Comprehensive Soldier and Family Fitness (CSF2) program, for example, focuses on building resilience, performance, and readiness through “dimensions of strength” that include exercise, nutrition, training, and self-control [47–48]. While there is limited scientific evidence available on this positive psychology approach in a military population, future research may identify targeted areas for refinement for ensuring optimal implementation [49–50].

While key strengths of this analysis included the longitudinal design for examining personnel in three different Army specializations and the ability to mutually adjust for multiple covariates, there were some notable limitations. It is possible that our sample may not be representative of all SF personnel, RQ infantrymen, and GPF infantrymen. However, the Millennium Cohort Study is based on a random sampling of all U.S. military members and investigation of potential sources of selection bias revealed a representative military population [51–54]. Since we relied on self-administered surveys, the information collected may be susceptible to recall errors. SF personnel and RQ infantrymen may be less inclined to report mental health issues for fear of stigma or that they will be removed from duty, although evidence suggests that all three groups may be similar in this regard [55–56]. In addition, we assessed other health outcomes, which may be less susceptible to stigma and reporting bias, and these outcomes demonstrated similar patterns. As with any prospective cohort study, loss to follow-up may potentially bias the results, although analyses on weighting for nonresponses have not identified changes in metrics for PTSD, depression, and disordered eating [57].

In summary, we found that SF personnel and RQ infantrymen were less likely to report mental health problems, physical health problems, and unhealthy behaviors than GPF infantrymen. Engaging in more healthy behaviors at baseline was associated with decreased odds for mental health and physical health problems at follow-up, regardless of Army specialization. This specific finding is of great importance to the military community as it indicates that engagement in modifiable healthy behaviors, independent of baseline health status, may significantly impact health outcomes. These findings suggest that further individual, unit, and leader encouragement of the adoption of healthy behaviors may be an efficient and cost-effective approach for preventing adverse health outcomes in military populations.
